# Zinc transporter-1 concentrates at the postsynaptic density of hippocampal synapses

**DOI:** 10.1186/1756-6606-7-16

**Published:** 2014-03-07

**Authors:** Carlos Sindreu, Àlex Bayés, Xavier Altafaj, Jeús Pérez-Clausell

**Affiliations:** 1 Department of Pharmacology, University of Barcelona, Barcelona 08036, Spain; 2 Department of Pharmacology, University of Washington, Seattle 98195, USA; 3 Molecular Physiology of the Synapse Laboratory, Biomedical Research Institute Sant Pau, 08025 Barcelona, Spain; 4 Universitat Autònoma de Barcelona, 08193 Bellaterra, Spain; 5 Biomedical Research Institute Bellvitge, L’Hospitalet de Llobregat 08908, Spain; 6 Department of Cell Biology, University of Barcelona, Barcelona 08028, Spain

**Keywords:** ZnT1, Hippocampus, Postsynaptic density, Vesicular Zn^2+^, PDZ I motif, Dendritic spine

## Abstract

**Background:**

Zinc concentrates at excitatory synapses, both at the postsynaptic density and in a subset of glutamatergic boutons. Zinc can modulate synaptic plasticity, memory formation and nociception by regulating transmitter receptors and signal transduction pathways. Also, intracellular zinc accumulation is a hallmark of degenerating neurons in several neurological disorders. To date, no single zinc extrusion mechanism has been directly localized to synapses. Based on the presence of a canonical PDZ I motif in the Zinc Transporter-1 protein (ZnT1), we hypothesized that ZnT1 may be targeted to synaptic compartments for local control of cytosolic zinc. Using our previously developed protocol for the co-localization of reactive zinc and synaptic proteins, we further asked if ZnT1 expression correlates with presynaptic zinc content in individual synapses.

**Findings:**

Here we demonstrate that ZnT1 is a plasma membrane protein that is enriched in dendritic spines and in biochemically isolated synaptic membranes. Hippocampal CA1 synapses labelled by postembedding immunogold showed over a 5-fold increase in ZnT1 concentration at synaptic junctions compared with extrasynaptic membranes. Subsynaptic analysis revealed a peak ZnT1 density on the postsynaptic side of the synapse, < 10 nm away from the postsynaptic membrane. ZnT1 was found in the vast majority of excitatory synapses regardless of the presence of vesicular zinc in presynaptic boutons.

**Conclusions:**

Our study has identified ZnT1 as a novel postsynaptic density protein, and it may help elucidate the role of zinc homeostasis in synaptic function and disease.

## Background

Homeostasis of ionic or labile zinc (Zn^2+^) in central neurons might be important in a range of physiological and pathological events. Zn^2+^ may act as a co-transmitter at certain glutamatergic synapses, participate in neuronal signal transduction, modulate memory formation and nociception, or promote neurodegeneration upon brain insults [1,2]. Marked differences in the levels of intracellular Zn^2+^ are found among cellular compartments owing to the coordinated actions of two families of zinc transporter proteins, Slc30a (ZnT1-10) and Slc39 (ZIP1-14). Whereas ZnTs export Zn^2+^ away from the cytosol into organelles or the extracellular space, ZIPs shuttle Zn^2+^ in opposite direction [3]. Cytosolic Zn^2+^ is estimated to be in the subnanomolar range, but Zn^2+^ transients in neurons have been reported following strong depolarization or oxidation [4]. Accumulation of cytosolic Zn^2+^ is common in degenerating neurons in models of epilepsy, ischemia or Parkinson’s disease [5-7]. In contrast, high concentrations of zinc are normally found at synapses [8]. Bound zinc maintains the organization of the postsynaptic density (PSD) [9], where it associates with Shank2/3 protein scaffolds [10] and SAP-102 [11]. In addition, a subset of excitatory boutons up-take Zn^2+^ into glutamatergic vesicles via ZnT3 [12]. One may expect, therefore, that specific plasma membrane proteins support Zn^2+^ homeostasis at synapses, but their identity remains elusive. One candidate protein is ZnT1 [13]. ZnT1 localizes to the plasma membrane, reduces cytosolic Zn^2+^, confers resistance against Zn^2+^ toxicity, and it is expressed in several brain regions [13,14]. We previously developed a protocol that allows for the co-localization of neuronal proteins and vesicular Zn^2+^ by combining immunogold electron microscopy with zinc histochemistry [15]. Here we used a similar approach to ask whether ZnT1 localizes to synapses. We focused on the CA1 region of the hippocampus because only half of CA3-to-CA1 synapses contain vesicular Zn^2+^[15], allowing for direct comparisons between the presence of vesicular Zn^2+^ and ZnT1 expression.

## Results and discussion

### ZnT1 is found in synaptic regions in hippocampus

**Figure 1:**
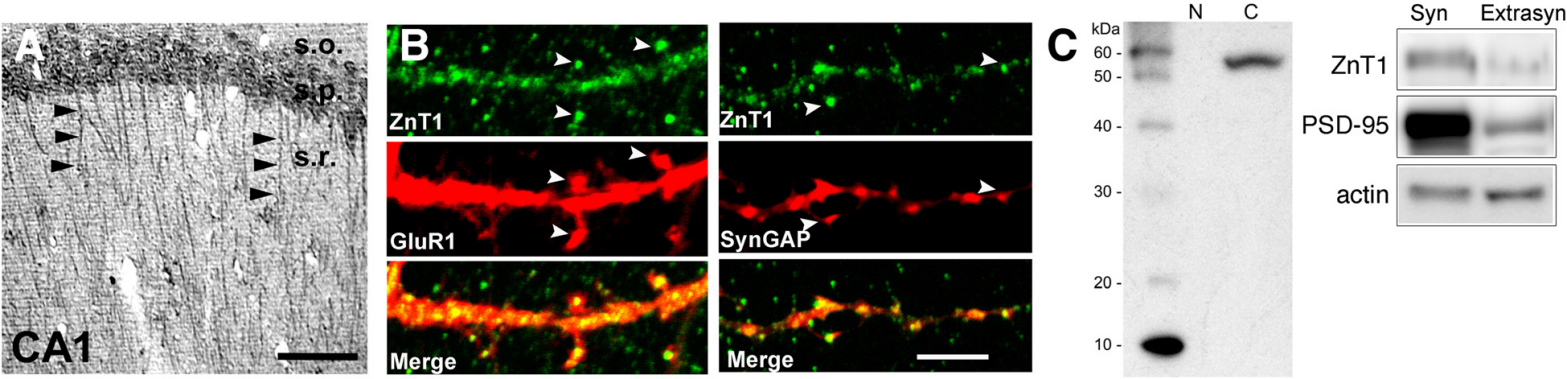
**Synaptic targeting of ZnT1. (A)** Bright field immunostaining of ZnT1 in mouse CA1 region. Neuronal perikarya and apical dendrites (arrowheads) were labeled. s.o. stratum oriens; s.p. stratum piramidale; s.r. stratum radiatum. Scale bar, 100 μm. **(B)** Confocal images of double stained dendrites of cultured pyramidal neurons. Scale bar, 5 μm. **(C)** Left, western blot for ZnT1 in nuclear (N) and cytoplasmic **(C)** extracts (20 μg per lane) from adult hippocampus. MW was assessed with biotinylated protein ladder. Right, Synaptic and extrasynaptic fractions (10 μg per lane) were probed for ZnT1, stripped, and reprobed for actin and PSD-95.

Immunostaining for ZnT1 in the CA1 region of the hippocampus was particularly conspicuous in somata and apical dendrites of pyramidal cells (Figure 1A), prompting us to analyze its synaptic distribution. In mature hippocampal cultures (DIV 21), ZnT1 co-localized with GluR1(+) and SynGAP(+) puncta along dendritic shafts (Figure 1B), indicating the presence of ZnT1 in spines. As predicted, ZnT1 appeared as a 55 kDa band in the cytoplasmic fraction of hippocampal lysates (Figure 1C). When synaptic and extrasynaptic membranes were separated, ZnT1 was enriched in the synaptic (i.e. Triton-insoluble and PSD95-rich) plasma membrane fraction (Figure 1C). The presence of ZnT1 at synapses was independently confirmed by mass spectrometry-based analysis of adult mouse brain synaptosomal fractions (Bayés A, personal communication).

### ZnT1 concentrates at the postsynaptic density

**Figure 2:**
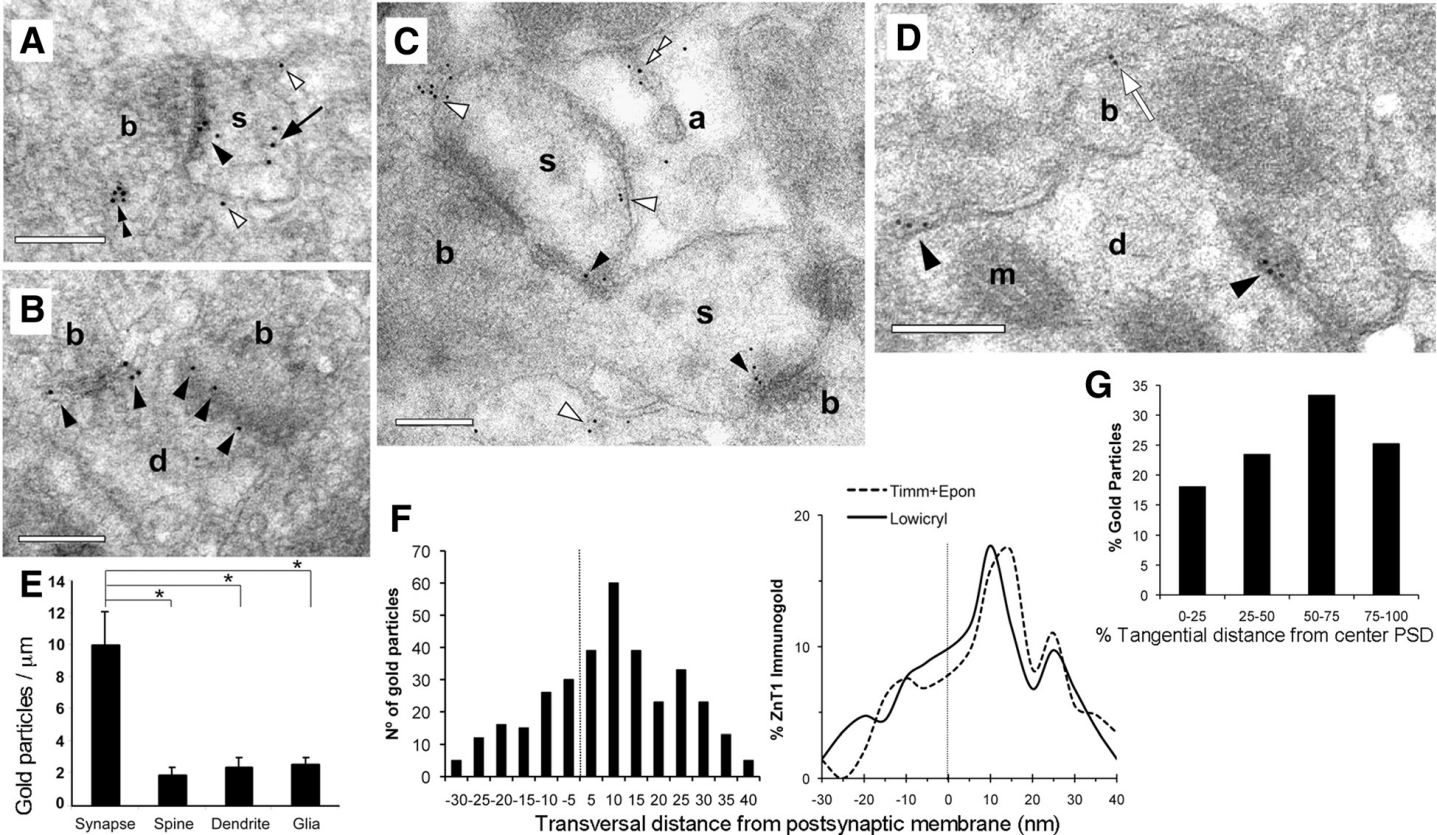
**Ultrastructural localization of ZnT1.** Electron micrographs of Lowicryl sections showing ZnT1 particles (10 nm gold) at synapses made on dendritic spines **(A, C)** or shafts **(B, D)**. Particles predominated over synaptic junctions (solid arrowheads). Extrasynaptic labeling was seen over the plasma membrane of spines and dendrites (white arrowheads). Occasional labeling on extrasynaptic membranes apposed to boutons (white arrow in **D**), inside astrocytes (double white arrowhead in **C**), inside spines (solid arrow in **A**), or over presynaptic vesicles (double solid arrowhead in **A**). b, bouton; s, spine; d, dendrite; a, astrocyte; m, mitochondrion. Scale bars, 0.2 μm. **(E)** Surface densities of ZnT1 (particles/μm) over different plasma membranes. **(F)** Left, Frequency distribution of gold particles across the synaptic junction (i.e. axodendritic). Negative and positive values denote locations pre- and post-synaptic to the outer leaflet of the postsynaptic membrane, respectively. Distances between the center of gold particle and membrane were grouped into 5 nm bins. Particles followed a normal distribution with a peak located at the +10 nm bin. Right, Relative distribution of ZnT1 across the synaptic junction analyzed in Lowicryl and Epon sections. **(G)** Relative distribution of ZnT1 along the PSD length. Particle location was computed as a fraction of the distance from the center (0%) to the edge (100%) of the PSD.

We first established the ultrastructural distribution of ZnT1 in Lowicryl-embedded CA1 stratum radiatum in the absence of zinc histochemistry. Postembedding immunogold showed that 80 ± 1% of ZnT1 particles localized to the plasma membrane, including synaptic junctions, dendrites, and astrocytes (n = 1,372 gold particles; area analyzed = 354 μm^2^; Figures 2A-D). ZnT1 particles often accumulated at membrane densities of apposed plasma membranes, including synaptic junctions or presumed puncta adherentia. Indeed, quantitative analysis revealed that the ZnT1 surface density was > 5-fold higher in synaptic membranes compared with extrasynaptic membranes in spines, dendritic shafts, or glia (n = 334 particles over 88.4 μm of membrane; Figure 2E; P < 0.001 for all comparisons). In the absence of primary antibody, the density of synaptic particles was markedly lower (0.04 ± 0.03 particles/μm). The small fraction of intracellular ZnT1 labeling was mainly associated with postsynaptic membranous material (67 ± 2% of intracellular particles) (Figure 2A).

Synaptic ZnT1 labeling was widespread, encompassing 76 ± 3% of asymmetric synapses in single ultrathin sections (3.2 ± 1.7 particles/synapse; range 1–9; n = 175 synapses). Particle distribution along the axis perpendicular to the synaptic junction revealed a main peak on the cytoplasmic side of the postsynaptic membrane (7.1 ± 0.8 nm from the membrane; n = 339 particles and 132 synapses) (Figure 2F). Further, tangential analysis of synaptic ZnT1 revealed a moderate lateral increase along the PSD length (n = 111 particles and 46 synapses; Figure 2G). The results indicate that ZnT1 concentrates in the postsynaptic membrane of hippocampal synapses, in keeping with the biochemical data.

### Synaptic ZnT1 expression and vesicular Zn^2+^

**Figure 3:**
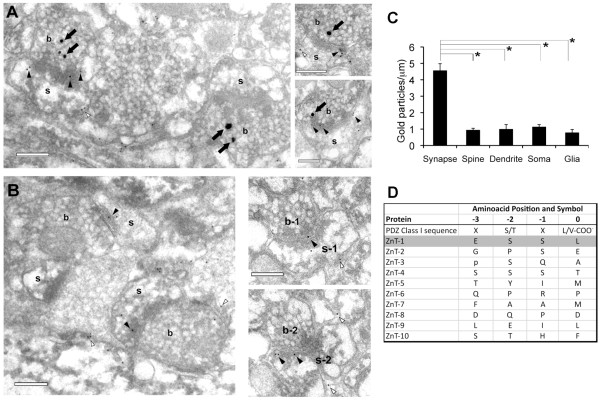
**Co-labeling of ZnT1 and vesicular Zn**^**2+**^**. (A)** Sections of CA1 synapses stained for vesicular Zn^2+^ (solid arrows) and ZnT1 (solid arrowheads, synaptic; white arrowheads, extrasynaptic). **(B)** ZN- synapses labeled for ZnT1 at the synaptic junction. Right, Consecutive sections of a ZN- axospinous synapse labeled for ZnT1. b, bouton; s, spine. Scale bars, 0.2 μm. **(C)** Surface density of gold particles showing the accumulation of ZnT1 in synaptic junctions (i.e. ZN + and ZN- synapses pooled) compared with various extrasynaptic membrane domains. **(D)** Sequence analysis of ZnT members showing that only ZnT1 contains a canonical PDZ type I motif in its C-terminus.

To determine if synaptic ZnT1 expression correlates with the presence (ZN+) or absence (ZN-) of vesicular Zn^2+^ in presynaptic boutons, we performed zinc histochemistry before tissue embedding. Previous studies have shown that ZN + synapses selectively co-express the obligatory vesicular transporter ZnT3, and make up ~45% of axospinous synapses in CA1 [15]. Indeed, silver granules reporting vesicular Zn^2+^ were invariably found in a fraction of boutons and readily distinguished from immunogold particles (Figure 3A). As earlier, ZnT1 particles were significantly enriched in synaptic junctions (area analyzed = 316 μm^2^; membrane length = 285.4 μm; n = 522 particles; Figure 3C), and accumulated just inside the postsynaptic membrane (9.2 ± 1.2 nm; 145 particles; Figure 2F). Synaptic ZnT1 labeling was found with similar frequencies at ZN + and ZN- axospinous synapses in single sections (58 ± 1% of ZN + synapses, and 60 ± 3% of ZN- synapses; n = 143 synapses; P = 0.9; Figure 3A, B). Likewise, the number of synaptic ZnT1 particles was similar between both populations (2.2 ± 0.2 particles/PSD at ZN+, and 1.5 ± 0.8 particles/PSD at ZN-; n = 214 particles; P = 0.2). Serial section analysis confirmed the absence of vesicular Zn^2+^ in synapses labeled for ZnT1 that had been identified as ZN- in single sections (n = 51; Figure 3B, right). Moreover, although distal segments of stratum radiatum are known to stain for Zn^2+^ more heavily [12], we found similar ZnT1 densities at proximal (< 20 μm from soma) and distal (> 100 μm away) synapses (n = 103 and 199 synapses, respectively; P = 0.2). We conclude that postsynaptic ZnT1 expression does not correlate with vesicular Zn^2+^ content in individual synapses.

The present results provide compelling evidence that ZnT1 is preferentially expressed in synaptic membrane domains in rodent hippocampus (mouse and rat), and identify ZnT1 as a novel PSD protein. ZnT1 was found in the somatodendritic compartment in region CA1, it co-localized with PSD proteins GluR1 and SynGAP in dendritic spine-like structures, and it was enriched in synaptic membrane fractions compared with extrasynaptic membrane. High-resolution immunogold electron microscopy confirmed and extended these results, showing that ZnT1 densities were over 5-fold higher in postsynaptic junction membranes than in other membrane domains, including extrasynaptic spine membrane, shafts, boutons, somata or astrocytes. Furthermore, the ZnT1 particle distribution across the synapse, peaking within 10 nm inside the postsynaptic membrane, suggests that ZnT1 is an integral transmembrane protein at the PSD, which is consistent with the predicted 6 hydrophobic regions in its aminoacid sequence [13]. The fact that ZnT1 surface density was similarly low among several extrasynaptic regions suggests that, in addition to the secretory pathway targeting ZnT1 to the cell surface, a synapse-specific mechanism may retain ZnT1 at the PSD. One possibility is that ZnT1 interacts via its cytoplasmic regions with other PSD proteins to concentrate at synapses. Interestingly, ZnT1 is the only member of its family that presents a consensus PDZ Class I motif at its C-terminus (Figure 3D), the latter showing 100% identity in humans. Several scaffolding molecules recognize PDZ motifs, including PSD-95, MPP1 or Lin7. Since the ZnT1 PDZ motif (ESSL) is very similar to that of GluN2A/B (ESDV), one may speculate that proteins of the PSD-95 family may also be retaining ZnT1 at the PSD.

Notably, ZnT1 was not restricted to the subset of synapses containing vesicular Zn^2+^. This widespread distribution implies a general physiological role for ZnT1 at synapses independently of the actions of ZnT3 or vesicular Zn^2+^. Accordingly, organizing synaptic scaffolds appear particularly sensitive to intracellular Zn^2+^, leading to changes in PSD morphology upon zinc chelation [16]. It is therefore possible that zinc status itself affects ZnT1 localization or expression. ZnT1 may be responsible for counteracting basal ZIP-mediated influx of interstitial Zn^2+^[17] or for removing Zn^2+^ liberated in dendrites from redox-sensitive metalloproteins, such as metallothionein-3 or PKC [18,19]. In keeping with this, deletion of metallothionein-3 prevents the build-up of cytosolic Zn^2+^ following seizures [20]. Additional roles for ZnT1 besides Zn^2+^ transport, such as modulation of MAPK or Ca^2+^ signaling [21,22], or targeting of potential PSD protein partners, cannot be ruled out. Genetic deletion of ZnT1 is embryonic lethal [23]. Given the high concentration of elemental zinc and ZnT1 at PSDs, it will be interesting to learn how conditional deletion of ZnT1 affects synaptic structure and function.

## Conclusion

ZnT1 is the first zinc transporter protein shown to concentrate at the PSD. The presence of ZnT1 in the vast majority of excitatory synapses in hippocampus suggests an unanticipated basic role for ZnT1 in synaptic function that warrants further investigation.

## Materials and methods

### Antibodies

Rabbit anti-ZnT1 antibodies were obtained from William F. Silverman (Ben-Gurion University). The specificity of the antibody, raised against the carboxyl-tail of mouse ZnT1 [14], was previously demonstrated by transfection of HEK-293 cells with mouse ZnT1 plasmid, and by siRNA-mediated knockdown in rat cortical neurons [24]. Rabbit anti-GluR1 antibody, mouse anti-PSD95, and mouse anti-actin were from Millipore; rabbit anti-SynGAP was from Affinity Bioreagents.

### Immunohistochemistry

All procedures followed present regulations for animal care and handling and were approved by the University of Barcelona Ethical Committee. BALB/c mice (2 month-old) were anesthesized and perfused with heparinized saline, and 4% formaldehyde/0.1% glutaraldehyde in 0.1 M phosphate buffer (PB; pH 7.4). Brains were postfixed for 2 hr, cryoprotected in 30% sucrose in PB, frozen, and cut into 40 μm-thick sections. Brain slices were freeze-thawed twice with liquid N_2_, rinsed in PB, incubated in 0.5% NaBH_4_ in PB for 15 min, and blocked in 10% NGS, 1% BSA and 0.2 M glycine in PBS for 2 hr. Slices were incubated in rabbit anti-ZnT1 antibody (1:500) at 4°C for 2 d, then rinsed and incubated in biotinylated goat anti-rabbit IgG (1:200) at 4°C for 8 hr, rinsed again, and incubated in streptavidin-biotinylated HRP (1:300). The HRP was developed in 0.05% DAB and 0.001% H2O2 in Tris–HCl buffer.

### Neuronal cultures

Hippocampi were collected from postnatal day 1 pups, digested with 10 U/ml papain in Hank’s buffer, dissociated, and plated in Neurobasal A with supplements on poly-D-lysine-coated coverslips. Cultures were fixed with 4% formaldehyde, 4% sucrose in PB, and then 100% methanol for 10 min at 4°C. Tyramide-Cyanine 3 signal amplification was used according to manufacturer instructions (Perkin Elmer) to detect binding of anti-GluR1 (1:6,000) or anti-SynGAP (1:8,000) antibodies. Immunolabeling with anti-ZnT1 antibodies (1:500) was detected with goat anti-rabbit Alexa488 IgG. Images were captured using an Olympus Fluoview FV600 confocal microscope and x63 objectives. Z-series of 4 consecutive optical sections (0.6 μm) were acquired using sequential scanning and Kalman filter.

### Immunoblotting

Hippocampi were homogenized in 10 volumes of buffer containing 10 mM HEPES, pH 7.5, 0.32 M sucrose, 2 mM EDTA, 2 mM EGTA, 5 mM DTT, and protease inhibitor cocktail tablet (Roche), followed by centrifugation at 800 g for 10 min. Supernatants were saved as the cytoplamic fraction. Pellets were resuspended in homogenization buffer plus 0.5% Nonidet P-40, and centrifuged again to obtain crude nuclear fractions. These nuclear fractions were resuspended in extraction buffer containing 10 mM HEPES, pH 7.5, 400 mM NaCl, 10% glycerol, 0.5% Nonidet P-40, 2 mM EDTA, 2 mM EGTA, 5 mM DTT, and protease inhibitor cocktail. Suspensions were rocked for 30 min on ice and centrifuged at 16,700 g for 10 min. Resulting supernatants were used as nuclear extracts. The cytoplasmic fraction was centrifuged (10,200 g, 15 min) to obtain the crude synaptosomal pellet, resuspended with 0.5% Triton, rocked, and centrifuged at 21,000 g for 40 min. The PSD-enriched pellet was resuspended in LB. The supernatant containing non-PSD membranes was precipitated with -20°C acetone. Equal protein amounts were separated by SDS-PAGE in 8% gels. PVDF membranes were blocked with 5% BSA, 0.1% Tween-20 in Tris-buffered saline (TBS), and incubated overnight at 4°C with ZnT1 antibodies (1:1,000), or 1 h at RT with PSD-95 (1:10,000) or actin (1:5,000) antibodies. Proteins were detected with HRP-conjugated secondary antibodies and visualized by chemiluminescence.

### Post-embedding immunoelectron microscopy

For acrylic embedding, brains from two Wistar rats (250–300 g) were perfused with 4% formaldehyde, 0.2% glutaraldehyde and 0.2% picric acid in PB, pH 7.4. Vibratome slices were cryoprotected in glycerol, frozen in liquid propane, freeze-substituted in 0.5% uranyl acetate in methanol at -90°C, infiltrated with Lowicryl HM20 at -45°C, and polymerized with UV light. For Zn^2+^ staining and epoxy embedding, we used our previous protocol (Sindreu’03). Briefly, two rats were perfused with 0.1% Na_2_S and 2.5% glutaraldehyde in PB, followed by 1% formaldehyde, 2.5% glutaraldehyde and 0.2% picric acid. Slices were stained with the Neo-Timm’s method (Danscher, 1981), post-fixed using an osmium-free protocol (Phend et al., 1995) and flat-embedded in epoxy resin (Embed-812; EMS). Lowicryl or epoxy sections (60–70 nm) were collected on gold grids (200 mesh), incubated in 0.5% NaBH_4_ in 50 mM Tris-buffered saline (TBS; pH = 7.6) for 10 min, rinsed in 0.005% Tergitol NP-10 in TBS (TBST), blocked in 10% NGS, 2% BSA and 50 mM glycine in TBST, and reacted with anti-ZnT1 antibodies (1:200) overnight. Grids were rinsed in TBST and TBST/pH 8.2, and incubated in 10 nm gold-conjugated goat anti-rabbit F(ab)_2_ fragments (1:30; British BioCell) for 2 h. Grids were rinsed and counterstained.

Electron micrographs were taken at × 30,000 magnification, recorded by a CCD camera (Gatan Inc.) and analysed with Canvas software (Deneba Systems, Inc.). The CA1 stratum radiatum was examined by random systematic sampling of visual fields (1/3) at 10–200 μm from the pyramidal cell layer. Synapses were defined as those structures showing synaptic vesicles in the presynaptic element and a postsynaptic membrane specialization. Labeling at the synaptic junction was analysed in transversely cut synaptic profiles. To assess the transversal location of synaptic gold particles, we measured the distance from the outer margin of the postsynaptic junctional membrane to the center of each gold particle in a line perpendicular to the membrane. Particles were considered ‘synaptic’ when found within a radius of 30 nm from the postsynaptic junctional membrane, and ‘extrasynaptic’ when located further away. Similarly, a 30 nm limit was used to differentiate between surface and intracellular labeling. To assess the tangential location of particles, the distance of each gold particle from the centre of the PSD was normalized to the half-length of the PSD profile. PSD lengths shorter than 200 nm were excluded for tangential analysis. To calculate the surface density, gold particles in each membrane compartment were counted in digital EM pictures, and effective membrane lengths measured. Double staining for vesicular Zn^2+^ and ZnT1 was studied in synapses located within 4 μm from the net surface of the vibratome slice to avoid false negative counts [15]. T-test and ANOVA were used for pair-wise and multiple comparisons, respectively. Significance was set at 0.05. Data are given as mean ± standard error of mean unless otherwise noted.

## Competing interests

The authors declare they have no competing interests.

## Authors’ contributions

CS performed experiments. CS, AB, XA and JPC analyzed the data. CS wrote the manuscript with input from all authors. All authors read and approved the final manuscript.
